# Supplementary material for: Selective Laser-Assisted Direct Synthesis of MoS2 for Graphene/MoS2 Schottky Junction
Source: Nanomaterials (Basel). 2023 Nov 13;13(22):2937. doi: 10.3390/nano13222937 (PMC10674199; doi:10.3390/nano13222937)

Supplementary Materials

Selective Laser-assisted Direct Synthesis of MoS_2 for Graphene/MoS_2 Schottky Junction

Min Ji Jeon[1], Seok-Ki Hyeong[2], Hee Yoon Jang[1], Jihun Mun[3], Tae-Wook Kim[4,5], Sukang Bae[2,5] and Seoung-Ki Lee[1*]*

[1]School of Material Science and Engineering Pusan National University, Busan 46241, Republic of Korea

[2]Institute of Advanced Composite Materials, Korea Institute of Science and Technology, Wanju, Jeonbuk 55324, Republic of Korea

[3]Advanced Instrumentation Institute, Korea Research Institute of Standards and Science, Daejeon 34113, Republic of Korea,

[4]Department of Flexible and Printable Electronics, Jeonbuk National University, Jeonbuk 54896, Republic of Korea

[5]Department of JBNU-KIST Industry-Academia Convergence Research, Jeonbuk National University, Jeonbuk 54896, Republic of Korea

Figure S1. Schematic diagram of Graphene/MoS_2 based optoelectronic device.

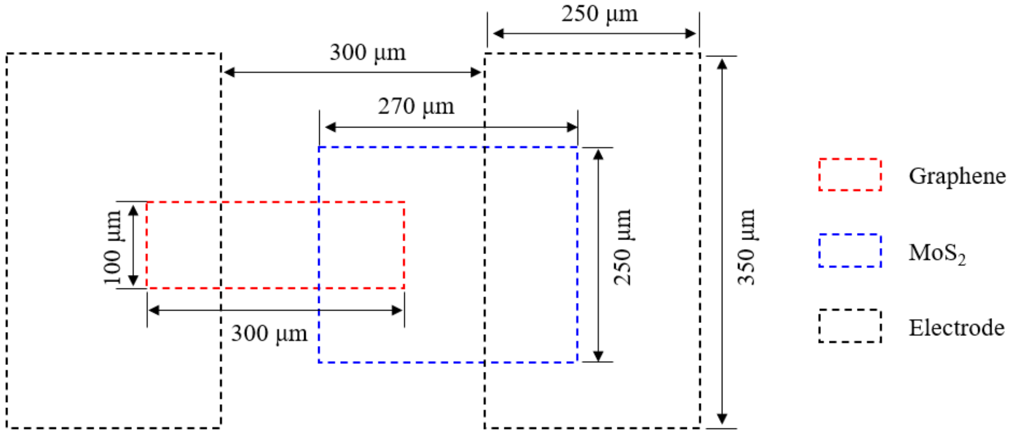

Graphene pattern: The graphene layer was patterned into dimensions of 100 μm by 300 μm to serve as the base for our heterostructure.

MoS_2 pattern: The MoS_2 layer was synthesized and patterned simultaneously by laser annealing to dimensions of 250 μm by 270 μm. The MoS_2 pattern was partially overlapped with the graphene pattern to form the Schottky barrier.

Electrode pattern size: Each electrode pattern was designed with dimensions of 250 μm by 350 μm to provide adequate contact area for electrical measurements without significantly overshadowing the active channel area. The distance between electrodes is 300 μm

Thickness of electrode materials: The electrodes are designed as a bilayer structure, consisting of a chromium adhesion layer of 5 nm, immediately followed by an 80 nm layer of gold. The chromium underlayer is essential for enhancing the adhesion of gold to the substrate. Nonetheless, the precise thickness of this chromium layer is critical to minimize contact resistance between MoS_2 and the metal contacts [S1]. Our experimental results indicate that a chromium layer thinner than 5 nm ($t < 5$ nm) is insufficient as an adhesion layer, whereas a thickness in excess of 10 nm adversely affects the uniformity of device performance. This non-uniformity is attributed to variable contact resistance between the MoS_2 and electrodes, likely a consequence of partial delamination of the electrode from MoS_2.[S1]

Reference

S1. Sano, K.; Takahashi, T.; Uchida, K. Large variability of contact resistance in Au/Cr/MoS2 system and its suppression by Cr thinning. Japanese journal of applied physics 2016, 55 (3), 036501.

Figure S2. AFM measurement

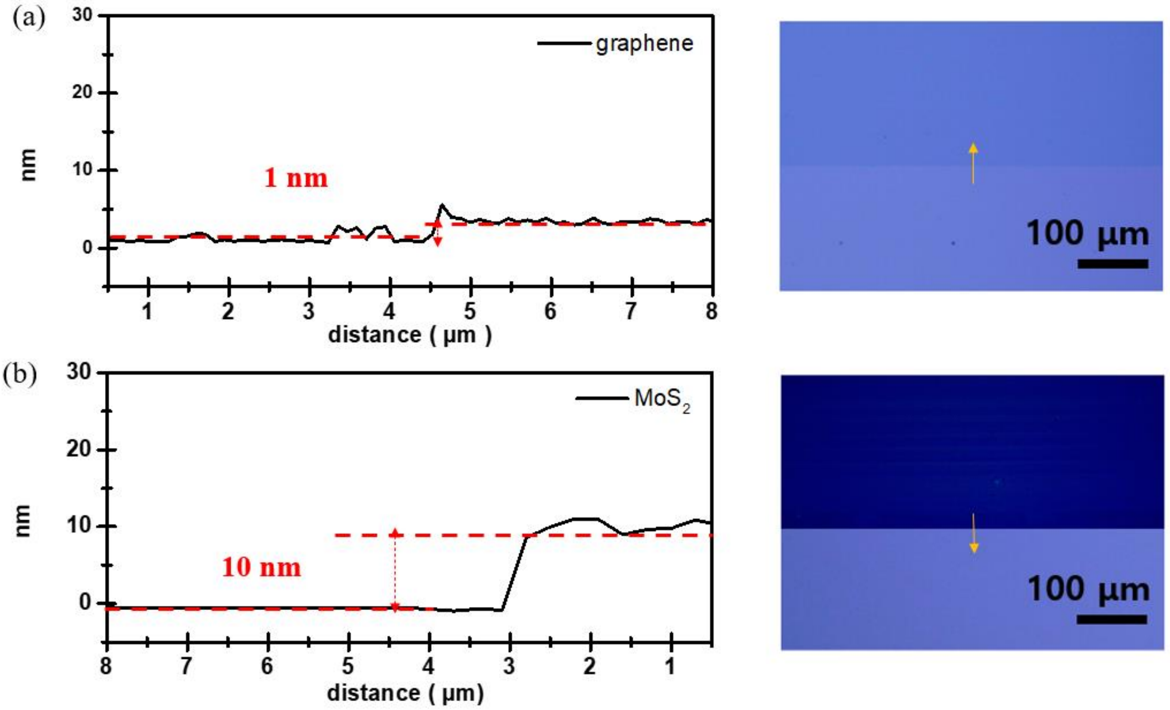

(a) Atomic force microscopy (AFM) height profile of the graphene (left) and optical microscope image (right) (b) height profile of the MoS_2 (left) and optical microscope image (right)

Figure S3. Transfer/output characteristics of MoS_2 FET

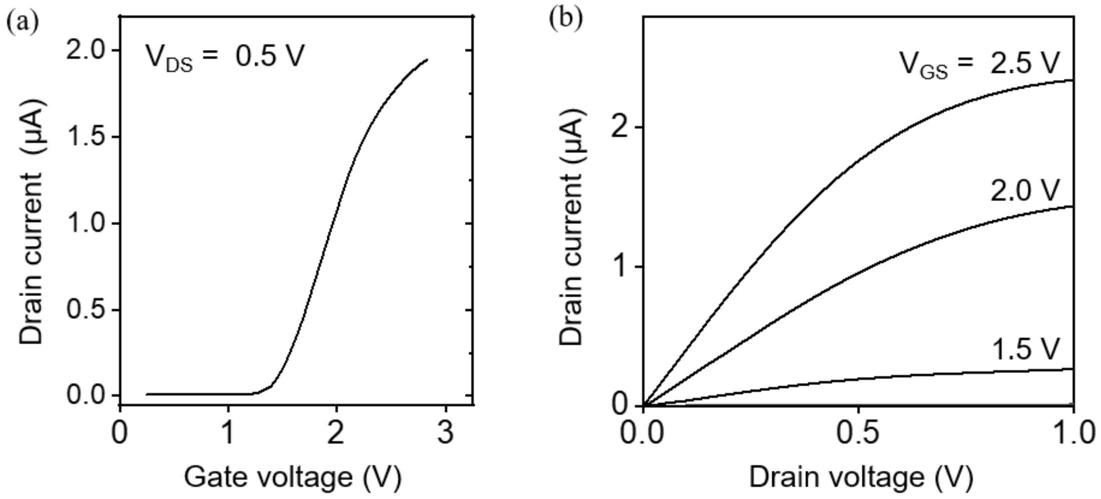

(a) Transfer characteristic and (b) output characteristic of Ion liquid gated MoS_2 FET.

Figure S4. Photocurrent Comparison between Graphene/MoS_2 and MoS_2/Cr/Au Junctions

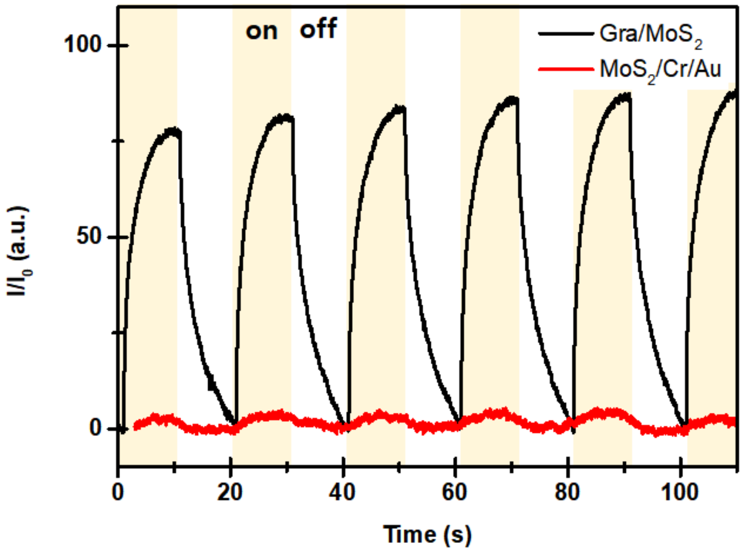

The incident laser used for the measurement possesses a wavelength of 473 nm, power of 3.5 mW, a pulse repetition rate of 50 mHz, and a variable spot size ranging from 50 to 200 µm.

Figure S5. Schematic diagram for manufacturing a CVD type device array

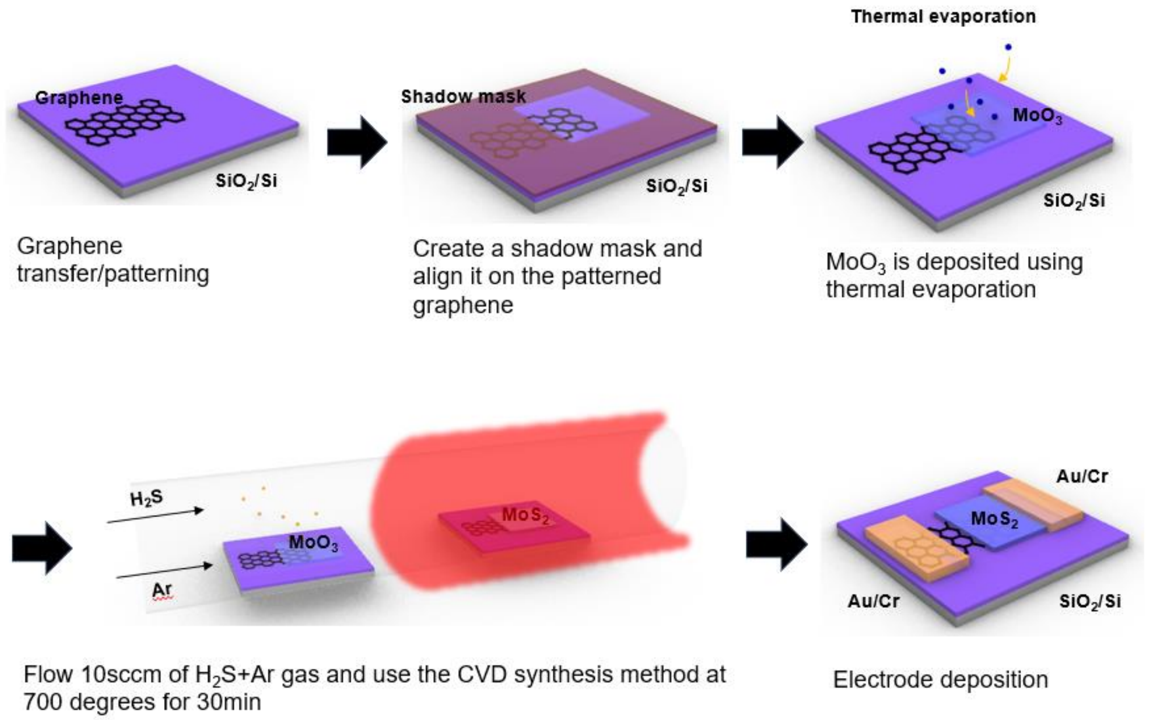

Figure S6. Photo-responsiveness of CVD type device

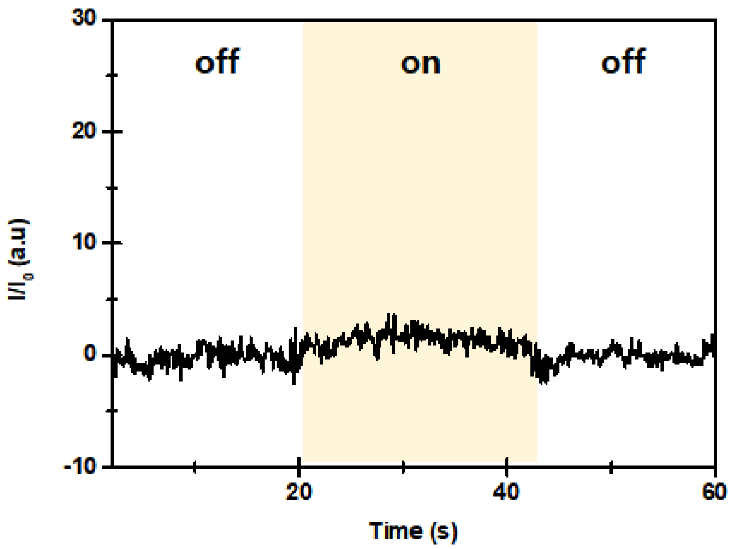

The CVD-type device has significantly lower reactivity to light than the laser photothermal-based device we used. In addition, damage to the lower layer graphene occurred due to the high temperature of 700 °C.

.

Figure S7. MoS_2 quality variation as a function of laser scanning speed

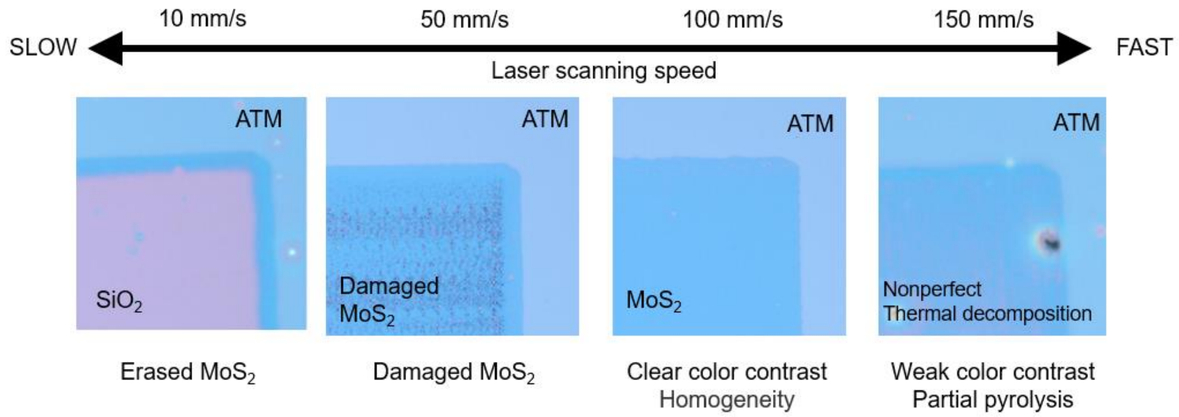

Figure S8. MoS_2 quality variation as a function of laser scanning speed

(a)

ATM amount (g)	M (mol/L)
1.0	0.633
1.4	0.887
1.8	1.140
2.0	1.270

(b)

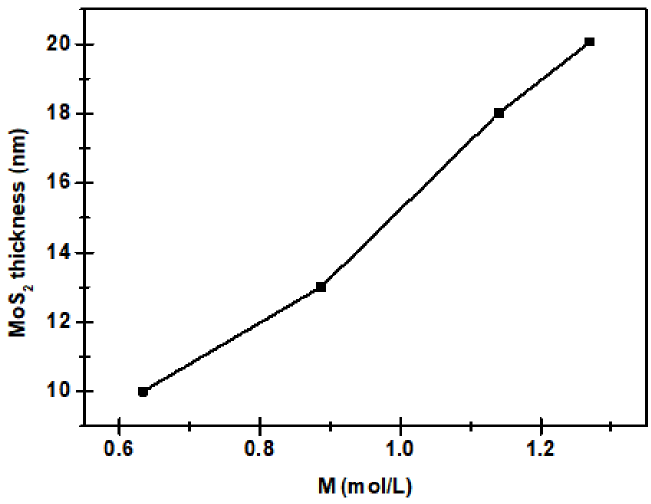

(a) Molal concentration according to mixed ATM mass (b) thickness of synthesized MoS_2 according to ATM precursor molal concentration

Figure S9. SEM image of MoS_2 boundary

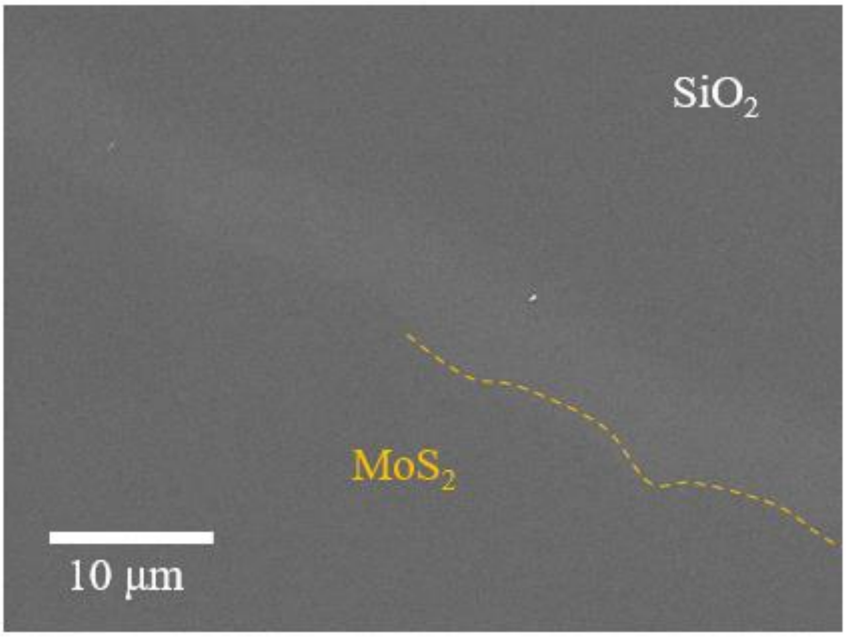

Supplement: Supplementary file 1 [file nanomaterials-13-02937-s001.zip › nanomaterials-2671088-supplementary.pdf]
